# Causal Relationship between Angina and Hepatic Failure as Revealed by Mendelian Randomization

**DOI:** 10.3390/jcm13020449

**Published:** 2024-01-13

**Authors:** Fengming Xu, Olaf Dirsch, Uta Dahmen

**Affiliations:** 1 Department of Infectious Diseases, The First Affiliated Hospital of Zhejiang Chinese Medical University, Hangzhou 310006, China; fengmingxu2020@gmail.com; 2 Else Kröner Graduate School for Medical Students “JSAM”, Jena University Hospital, 07747 Jena, Germany; 3 Institute of Pathology, Klinikum Chemnitz gGmbH, 09111 Chemnitz, Germany; olaf.dirsch@gmail.com; 4 Department of General, Visceral and Vascular Surgery, Jena University Hospital, 07747 Jena, Germany

**Keywords:** angina, hepatic failure, cardio-hepatic syndrome, risk factor, mendelian randomization

## Abstract

Background: Patients with angina are often suffering from comorbidities such as varying degrees of hepatic dysfunction. However, the impact of angina on the incidence of hepatic failure (HF) remains unclear. Methods: The genetic data were retrieved from genome-wide association studies. Five Mendelian randomization methods were used to investigate the causal relationship between unstable angina (UA), stable angina (SA), and HF. The result of the Inverse variance weighted (IVW) method was deemed the principal result. In addition, we performed a comprehensive sensitivity analysis to verify the robustness of the results. Results: The IVW results showed that UA (Odds ratio (OR): 2.055, 95% confidence interval (CI): 1.171–3.606, *p* = 0.012) was causally associated with the incidence of HF. SA (OR: 1.122, 95% CI: 0.738–1.706, *p* = 0.591) was not causally associated with the incidence of HF. Sensitivity analysis did not identify any bias in the results. Conclusions: UA turned out to be a risk factor for HF. SA does not have a significant causal effect on HF. Therefore, it is highly recommended that patients with chronic liver disease seek prompt medical attention and undergo regular monitoring of liver function when experiencing UA. This may help them to reduce the risk of HF.

## 1. Introduction

Both angina and hepatic failure (HF) are recognized as serious burdens on the health system and major causes of decreased quality of life and shorter life expectancy. Angina is one of the common symptoms of coronary artery disease (CAD) and usually occurs when the heart muscle is deprived of oxygen due to insufficient blood supply. This condition is usually caused by a narrowing or blockage of the coronary arteries [1]. Angina is currently a high-incidence disease, with a 2023 study from Sweden indicating an incidence of approximately 3.5% among middle-aged individuals in the general population [2]. The prevalence increases with age, making it more common in the elderly population [3].

Depending on the symptoms and characteristics of the attack, angina can be further differentiated into stable angina (SA) and unstable angina (UA). SA is typically triggered by vigorous physical activity or emotional excitement [4]. It is usually characterized by chest pain, possibly accompanied by chest tightness, pressure, or a feeling of throat constriction. These symptoms often quickly subside with rest or the use of medications like nitroglycerin. UA is a serious coronary condition that usually indicates unstable arterial plaque in the coronary arteries that may rupture and/or lead to the formation of intravascular blood clots. It is characterized by the unpredictability, instability, and severity of angina attacks. UA can strike at rest or with light physical activity, and UA pain is usually more intense, lasts longer, and is more difficult to relieve. If left untreated, it can lead to a myocardial infarction and heart failure [1,5,6].

Liver–heart interactions are multifaceted and have long been a topic of interest. In clinical practice, there is often a coexistence of cardiac and hepatic dysfunction in the context of common cardiac or hepatic diseases. Anatomically and physiologically, the liver and the heart are primarily interconnected via the “blood circulation”. Pathologically, heart disease can impact the liver, with examples including hepatic cirrhosis due to hepatic stasis, hypoxia, and connective tissue proliferation caused by recurrent episodes of chronic congestive heart failure or constrictive pericarditis [7,8,9]. Therefore, it is becoming increasingly important to determine the interactions between the heart and the liver to ensure the effective management of patients with heart or liver disease, thereby improving overall patient prognosis and treatment.

Currently, a less explored and intriguing facet of angina involves its potential to induce HF. HF is a critical condition where the liver loses its ability to perform its essential functions. The liver serves as a central metabolic organ responsible for tasks such as detoxifying the blood, processing nutrients, and synthesizing crucial proteins [10,11]. When the liver can no longer carry out these vital functions, it gives rise to a spectrum of distressing symptoms and complications such as jaundice, ascites, gastrointestinal bleeding, and hepatic encephalopathy [12,13,14], making it a daunting medical condition. A study from Germany in 2020 reported an overall mortality rate of 47% within three months among patients with acute HF [15]. As a severe liver disease with a high mortality rate, identifying risk factors of HF is of paramount importance in preventing the occurrence of HF.

HF may occur as a complication in patients with angina, especially in patients with pre-existing liver disease. Recent studies have proposed that systemic inflammation, which is elevated in angina patients, might affect the detoxification and protein metabolism functions of the liver, potentially contributing to HF [16,17,18]. In addition, it is believed that the severe and prolonged hypoxia associated with recurrent angina attacks can contribute to hepatic ischemia, subsequently causing hypoxic liver injury, also known as hypoxic hepatitis. Hypoxic hepatitis is characterized by centrilobular hepatocyte necrosis and a sharp elevation in serum aminotransferase levels. This may lead to a cascade of liver-damaging events culminating in HF [19,20,21]. Considering the potential underlying mechanisms mentioned above, we raised the hypothesis that UA and SA might be crucial risk factors for developing HF in humans.

Nowadays, Mendelian randomization (MR) has emerged as a powerful method for identifying causal relationships between risk factors and diseases via genetic variants [22,23,24]. Unlike observational studies, MR uses genetic variants randomly assigned at conception as instrumental variables (IVs) to estimate the causal effect of exposure on outcome and can lessen the bias due to confounders or reverse causation [25,26]. Therefore, this study applied two-sample MR (TSMR) analysis to identify a potential causal relationship between SA, UA, and HF.

## 2. Materials and Methods

### 2.1. Study Design

**Figure 1:**
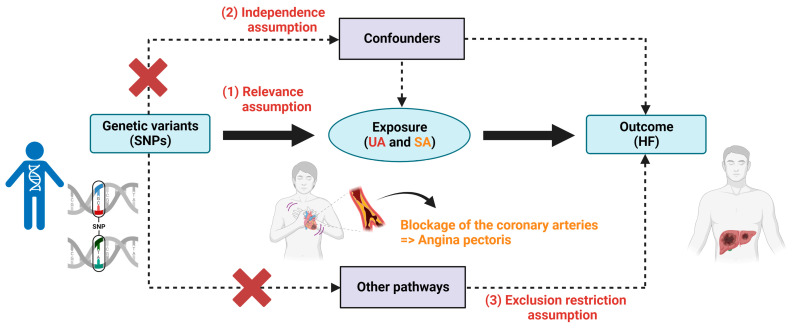
The causal relationship between UA, SA, and HF was explored by TSMR. The concepts and three core assumptions of the TSMR analysis are shown above. We created the figure with “Biorender.Com”.

In this study, we carefully explored the relationship between angina and HF via analyzing the role of two different degrees of angina (UA, representing severe angina, and SA, representing mild angina) in the incidence of HF. We analyzed the causal effect of UA and SA on the incidence of HF using TSMR. In our analysis, SA and UA were used as exposure factors and HF was used as an outcome measure. There are three core assumptions that need to be met to conduct the TSMR analysis: (1) the selected Single nucleotide polymorphisms (SNPs) should be significantly associated with exposure (UA, SA); (2) the selected SNPs should be independent of confounders; (3) the selected SNPs are associated with the outcome (HF) only via exposure (Figure 1). We used summary data from publicly available databases (OpenGWAS, Finnish Biobank) that had obtained participant consent and ethical approval (https://gwas.mrcieu.ac.uk/ (accessed on 19 October 2023)).

### 2.2. Data Source

SNPs for UA were extracted from a genome-wide association study (GWAS) dataset (ID: ebi-a-GCST90018932) including 9481 cases and 446,987 controls (sample size: 456,468) of European ancestry. SNPs for SA were extracted from a GWAS dataset (ID: ebi-a-GCST90018915) including 17,894 cases and 325,132 controls (sample size: 343,026) of European ancestry. Summary statistic data for HF (464 cases and 213,592 controls, sample size: 214,056) were obtained from a GWAS dataset (ID: finn-b-K11_HEPFAIL) of European ancestry. Specific summary information is shown in Table 1.

### 2.3. IV Selection Criteria

**Figure 2:**
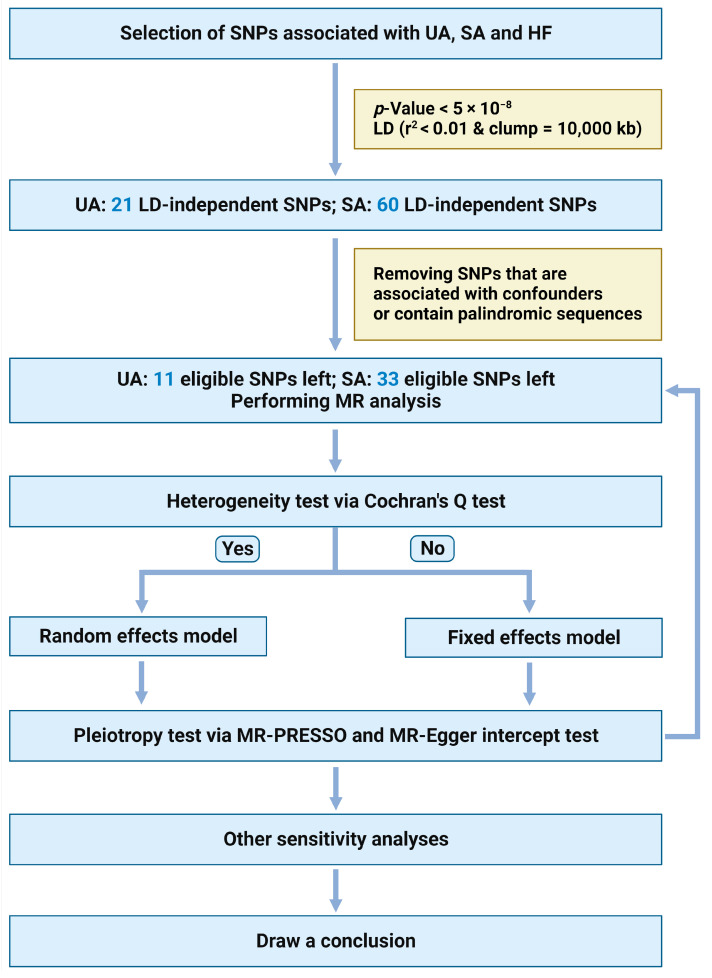
Flow diagram of the TSMR study. Abbreviations: MR, Mendelian randomization; LD, linkage disequilibrium; SNP, single nucleotide polymorphism. We created the figure with “Biorender.Com”.

We selected significant and independent SNPs for exposure factors (UA, SA) as IVs based on the following criteria:

(1) SNPs reached a genome-wide association significance level of *p* < 5 × 10^−8^.
(2) SNPs were independent, and the threshold of linkage disequilibrium (LD) was r^2^ < 0.01 and clumping window = 10,000 kb.
(3) SNPs with F-statistics < 10 were excluded to avoid weak instrumental bias, and the calculation formula is $F=\left(\frac{N-K-1}{K}\right)\left(\frac{R^{2}}{1-R^{2}}\right)$, where *R*^2^ represents the cumulative explained variance of the selected SNPs during exposure, *N* is the sample size of the exposure database, and *K* is the number of SNPs included in the analysis. F-statistics > 10 indicates a low likelihood of weak instrument bias.
(4) SNPs in the presence of palindromic sequence were excluded.
(5) SNPs associated (*p* < 1 × 10^−5^) with known confounders (e.g., Hepatitis A,B,C,D, and E, alcohol consumption, diabetes, obesity, autoimmune hepatitis, primary biliary cholangitis, primary sclerosing cholangitis, Wilson’s disease, rheumatoid arthritis, hypercholesteremia, taking cholesterol-lowering medication) were removed (Figure 2) [27].

SNP-related phenotypes were searched via a database of human genotype–phenotype associations (http://www.phenoscanner.medschl.cam.ac.uk/ (accessed on 19 October 2023)).

Following the rigorous screening criteria mentioned above, 11 SNPs were used as IVs to investigate the causal relationship between UA and HF (Table 2), while 33 SNPs were employed as IVs to study the causal relationship between SA and HF (Table 3).

### 2.4. MR Analysis

In this study, Inverse variance weighted (IVW), Weighted median, Weighted mode, Simple mode, and MR Egger methods were used to investigate the causal relationship between UA, SA, and the outcome HF. The IVW method assumes that all SNPs are valid IVs. The IVW method was used as the primary analysis method because of its high statistical power [28]. If the *p*-value of Cochran’s Q test is less than 0.05 (Table 4), the result of the MR analysis was analyzed using the IVW random-effects model; otherwise, the fixed-effects model was used. Weighted median, Weighted mode, Simple mode, and MR Egger methods were used as supplementary methods.

### 2.5. Sensitivity Analysis

Sensitivity analysis was performed to determine the robustness of the MR results. Cochran’s Q test was used to detect heterogeneity in the data, with a *p*-value of less than 0.05 indicating the presence of heterogeneity; MR-Pleiotropy RESidual Sum and Outlier (PRESSO) was used to detect horizontal pleiotropy and identify outliers. If outliers were found, they were removed and outlier-corrected MR analysis was performed to obtain an unbiased causal estimate. Directional pleiotropy was assessed via the MR-Egger-intercept test (Table 4). The leave-one-out analysis was used to assess whether MR results were strongly driven by specific SNPs. *p* < 0.05 was deemed as suggestive significance. The MR analysis was performed using the R package TwoSampleMR (version 0.5.7). The MR-PRESSO was conducted using the R package MR-PRESSO (version 1.0) in R program (version 4.3.1).

## 3. Results

### 3.1. UA Had a Positive Causal Effect on HF Risk

**Figure 3:**
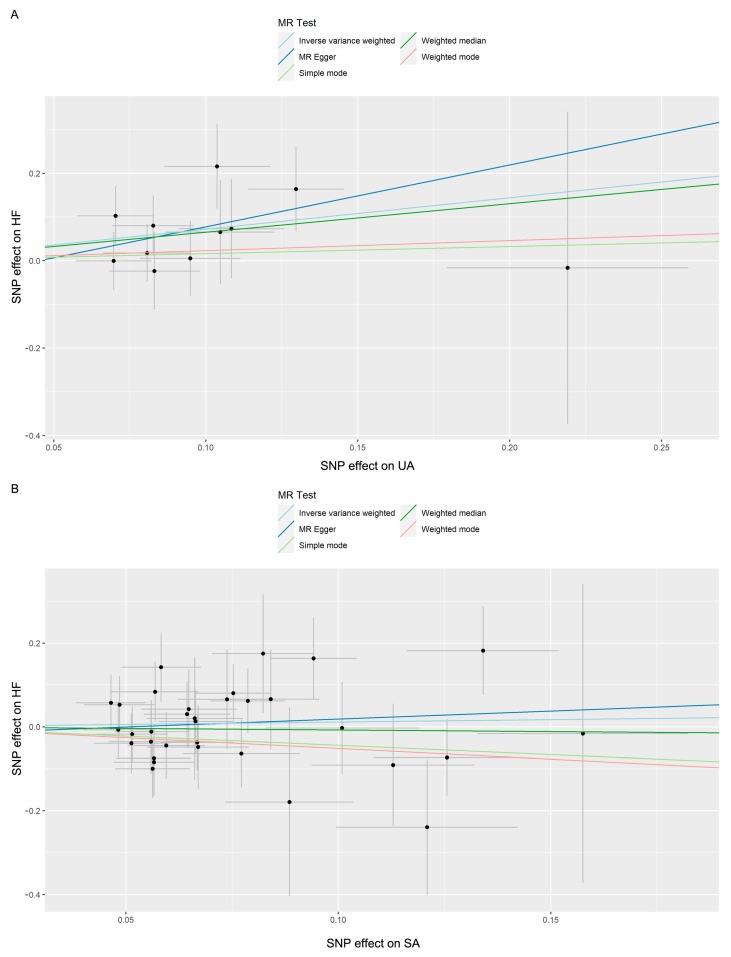
(**A**) Scatter plot demonstrating the causal relationship between UA and HF; (**B**) scatter plot demonstrating the causal relationship between SA and HF.

Using the 11 extracted SNPs that were eligible for IV screening (Table 2), we found a potential positive causal effect of UA on HF incidence. In the IVW analysis, UA was significantly correlated with HF (OR: 2.055, 95% CI: 1.171–3.606, *p* = 0.012). The results of the Weighted median (OR: 1.920, 95% CI: 0.901–4.092, *p* = 0.091), Weighted mode (OR: 1.258, 95% CI: 0.377–4.192, *p* = 0.717), Simple mode (OR: 1.177, 95% CI: 0.332–4.171, *p* = 0.806), and MR Egger (OR: 4.123, 95% CI: 0.305–55.721, *p* = 0.314) analyses were similar to the IVW analysis, but the results did not reach statistical significance. The estimated effect sizes of the SNPs on both UA and HF are displayed in scatter plots (Figure 3A).

**Figure 4:**
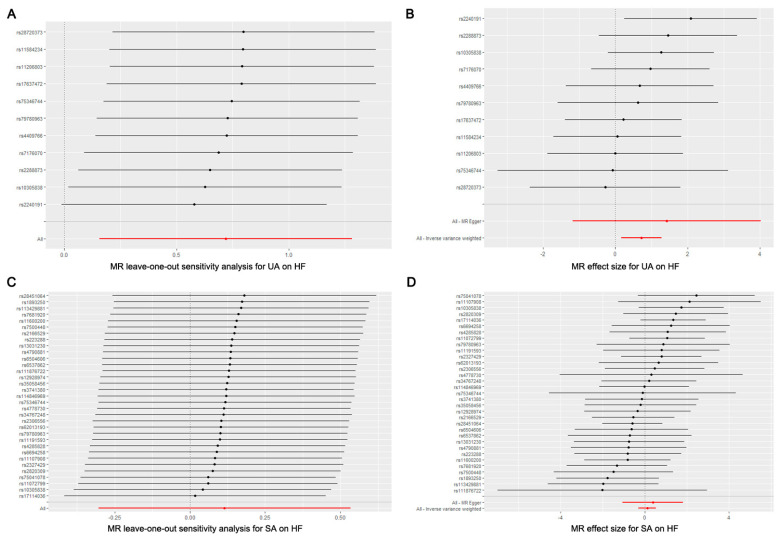
(**A**) Leave-one-out plot of SNPs associated with UA and HF; (**B**) forest plot of SNPs associated with UA and HF; (**C**) leave-one-out plot of SNPs associated with SA and HF; (**D**) forest plot of SNPs associated with SA and HF.

We calculated the F-statistic for each SNP, and the results were all greater than 10 (ranged from 30.458 to 68.246, Table 2), suggesting that there is a low likelihood of weak instrumental bias. Moreover, we used Cochrane’s Q test (IVW and MR Egger methods) to detect heterogeneity in the data. The results showed no significant heterogeneity with *p* = 0.821 > 0.05 for the IVW method and *p* = 0.775 > 0.05 for the MR Egger method. In addition, the result of the directional pleiotropy test by the Egger-intercept method was *p* = 0.604 > 0.05, indicating that the IVs did not significantly affect the outcome through pathways other than exposure. We used MR-PRESSO to confirm the absence of horizontal pleiotropy and outliers in the data (*p* = 0.827 > 0.05) (Table 4). Furthermore, no single SNP substantially violated the generalized effect of UA on HF incidence in the leave-one-out analysis, indicating that the results of the MR analysis were robust (Figure 4A). The forest plot showed that UA may increase the risk of HF (Figure 4B).

**Figure 5:**
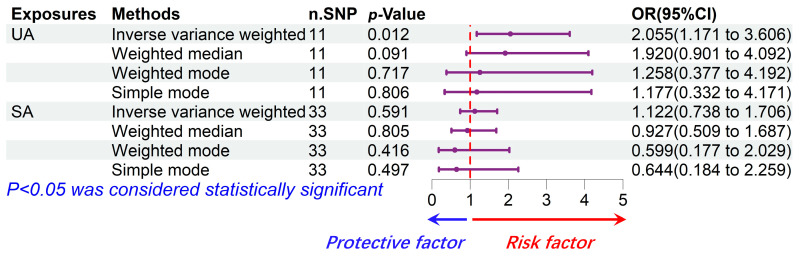
Forest plot for the causal relationship between UA, SA, and HF. Overall, UA is a risk factor for the incidence of HF and SA has no significant causal effect on the incidence of HF.

In summary, our results revealed that UA was positively associated with the risk of HF, suggesting that UA indeed might be a risk factor for the development of HF (Figure 5).

### 3.2. SA Had No Significant Causal Effect on HF Risk

Using the 33 extracted SNPs that were eligible for IV screening (Table 3), we found no significant causal effect of SA on HF incidence. In the IVW analysis, SA was not significantly correlated with HF (OR: 1.122, 95% CI: 0.738–1.706, *p* = 0.591). The result of MR Egger (OR: 1.463, 95% CI: 0.346–6.180, *p* = 0.609) analysis was similar to the IVW analysis. In contrast, the result of the Weighted median (OR: 0.927, 95% CI: 0.509–1.687, *p* = 0.805), Weighted mode (OR: 0.599, 95% CI: 0.177–2.029, *p* = 0.416), and Simple mode (OR: 0.644, 95% CI: 0.184–2.259, *p* = 0.497) differed from the above analysis, but the results did not reach statistical significance either. The estimated effect sizes of the SNPs on both SA and HF are displayed in scatter plots (Figure 3B).

We calculated the F-statistic for each SNP, and the results were all greater than 10 (ranged from 30.517 to 86.987, Table 3), suggesting that there is a low likelihood of weak instrumental bias. Using Cochrane’s Q test (IVW and MR Egger methods) revealed the absence of heterogeneity in the data (*p* = 0.770 > 0.05 for IVW method and *p* = 0.735 > 0.05 for MR Egger method). In addition, the directional pleiotropy test by the Egger-intercept method resulted in a *p*-value greater than 0.05 (*p* = 0.708 > 0.05), indicating that the IVs did not significantly affect the outcome through pathways other than exposure. MR-PRESSO analysis confirmed the absence of horizontal pleiotropy and outliers as well (*p* = 0.722 > 0.05) (Table 4). Furthermore, no single SNP substantially violated the generalized effect of SA on HF risk in the leave-one-out analysis, indicating that the results of the MR analysis were robust (Figure 4C). The forest plot showed that SA had no significant causal effect on HF risk (Figure 4D). 

In summary, our findings demonstrated that SA has no significant causal effect on HF. Nevertheless, the OR (1.122, 95% CI: 0.738–1.706) of SA calculated by the IVW method implies that SA may have some potential risk for HF (Figure 5).

## 4. Discussion

The role of angina in cardiovascular disease has been frequently discussed in the past. Recently, the impact of angina on liver function has been gaining more interest due to the introduction of the concept of “Cardio-hepatic syndrome” [29,30]. With the increasing research on the crosstalk between the heart and liver over the past decade, the term “Cardio-hepatic syndrome” nowadays refers to a syndrome characterized by clinical and laboratory manifestations of liver dysfunction in the presence of acute or chronic cardiac conditions [31]. Yet, relevant and reliable research evidence between angina and HF is scarce.

A clinical study from Austria that included 1002 study participants showed that hepatic dysfunction is prevalent in patients with chronic heart failure, and Poelzl et al. termed this medical condition as “Cardio-hepatic syndrome” [29,32]. In clinical practice, it is common to find cardiac disease patients with concurrent hepatic injury. The mechanisms by which cardiac dysfunction, e.g., due to CAD, leads to hepatic injury are usually related to hypoperfusion due to a reduced cardiac output and congestion secondary to volume overload [32].

In fact, the liver receives up to 25% of the cardiac output, making it highly sensitive to the reduction in blood flow [33,34]. A reduced blood flow due to CAD is one of the key causes of hypoxic hepatopathy. Hypoxic hepatitis, also known as “Ischemic hepatitis” or “Shock liver”, is an acute hepatic injury characterized histologically by the necrosis of hepatocytes in the center area of the lobule, caused by an insufficient oxygen supply to hepatocytes [35,36]. Affected by decreased cardiac function, patients with hypoxic hepatitis exhibit a higher central venous pressure (CVP) and lower hepatic blood flow. If hypoxic hepatitis is not intervened in a timely manner, it may lead to fulminant HF [37,38].

However, heart failure indicates a certain degree of loss of heart function and occurs in the severe stages of heart disease [39]. Heart failure patients with a lower ejection fraction tend to have a poorer prognosis [40]. The occurrence of angina is a typical manifestation of myocardial ischemia and is also a key node in determining cardiac function [41,42]. At this juncture, actively addressing the primary cardiac condition in patients may also have a positive impact on improving their liver function. Therefore, our carefully designed MR study aimed to clarify the impact of different stages of angina (SA and UA) on HF, which is of high value for improving the prognosis of patients with angina and underlying liver disease.

Our study is the first MR analysis to explore the role of angina in the pathogenesis of HF. In this TSMR analysis, we comprehensively explored the relationship between UA, SA, and HF. By analyzing a large amount of sample data, our results revealed that UA is indeed a risk factor for HF (OR: 2.055, *p* = 0.012), whereas SA does not appear to have a significant causal relationship with HF (OR: 1.122, *p* = 0.591). The results are in line with other studies using a completely different approach. Taken together, our study reveals a strong relationship between UA and HF. Our findings suggest a significant up-regulation of the risk of HF as the degree of angina continues to worsen.

MR studies also have some limitations. A primary limitation of MR is its applicability only to risk factors with suitable genetic variants. Genetic variants typically have a modest impact on most risk factors, which may result in a lower statistical power and the risk of false-negative results in MR analysis. To address this limitation, using multiple genetic variants associated with the risk factor as IVs can increase the proportion of variance explained, thereby enhancing statistical power [43], as we did in our MR study.

Our study has several major strengths:

First, we explored the causal relationship between UA, SA, and HF from a genetic perspective based on newly published (all datasets published in 2021) large-sample GWAS data (total sample size exceeds 1 million, Table 1). It is less susceptible to confounding factors and reverse causality than previous observational studies, making the research findings more reliable.

Second, it is a TSMR analysis that lends itself to causal inference. We established strict selection criteria for IVs. Only SNPs meeting the relevance, independence, and exclusion-restriction assumptions of the MR analysis could be selected as IVs.

Third, we used comprehensive analytic tests to detect IVs for heterogeneity and pleiotropy, and all IVs passed the heterogeneity and pleiotropy tests. In addition, we executed a series of powerful MR methods to analyze the causal relationships among UA, SA, and HF. 

However, there are several limitations to our study: 

First, not all MR analysis method results yielded valid causal relationships. However, most of the analysis methods yielded similar results. Since all IVs passed the heterogeneity and pleiotropy tests, we chose the results of the IVW method with highest test efficacy as the primary reference result [28].

Second, the source population of the dataset is European, which limits the applicability of the results to non-European populations. More research is needed in the future to validate the applicability of the results to other populations and ethnicities.

Third, the specific mechanism by which UA induces HF remains unclear. There are some potential links between these two conditions that have attracted the attention of researchers. One of the intriguing aspects is the role of systemic inflammation. In both angina and HF, systemic inflammation plays a latent role in the progression of the diseases. In the context of angina, inflammation within the coronary arteries may contribute to the buildup of atherosclerotic plaques, leading to reduced blood flow to the heart. In the context of the liver, systemic inflammation is a potential factor in the pathogenesis of decompensated hepatic cirrhosis and HF [44]. Recent studies have proposed that systemic inflammation, which is elevated in angina patients, might affect the detoxification and protein metabolism functions of the liver, potentially contributing to HF [16,17,18].

Another potential mechanism is that UA can lead to hypoxic hepatitis, resulting in extensive hepatocyte necrosis. Typically, hypoxic hepatitis is preceded by an acute event in which there is a sudden decrease in oxygen availability to the liver. The most common causes of hypoxic hepatitis are hemodynamic instability and a reduction in hepatic blood flow, and UA is one of the primary etiologies responsible for these symptoms [45]. The gradual increase in coronary artery plaques leads to a narrowing of the arterial lumen, resulting in reduced blood flow to the heart and the occurrence of myocardial ischemia. The further progression of coronary artery plaques can lead to sudden rupture, forming a thrombus that obstructs the artery, causing a sudden reduction in blood flow and thus triggering UA [46].

**Figure 6:**
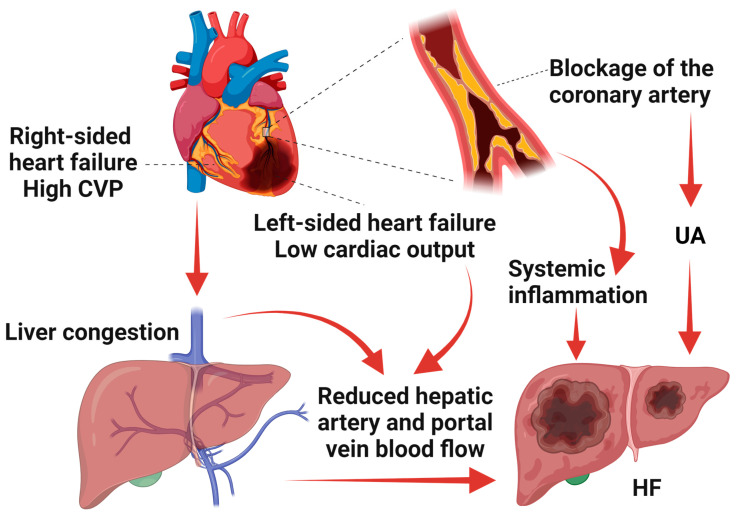
The results of TSMR showed that UA was a risk factor for HF. UA may cause HF by inducing a systemic inflammatory response, a reduced cardiac output, and a high CVP. We created the figure with “Biorender.Com”.

The third potential mechanism is that frequent UA leads to hepatic congestion, eventually inducing HF. Unstable angina is often accompanied by hemodynamic instability. Due to the absence of valves in the hepatic veins, increased inferior vena cava pressure can affect the sinusoidal bed, leading to sinusoidal dilation, central lobular congestion, and perivenous fibrosis. The major lesion occurs in the region surrounding the central veins. Hepatocellular necrosis in the central lobe may extend to the peripheral region, followed by the deposition and spread of connective tissue linking one central vein to another, eventually leading to cirrhosis and HF (Figure 6) [33,47,48]. Further exploration of the role of the UA-induced systemic inflammatory response, hypoxic hepatitis, and hepatic congestion in HF may help to clarify the link between UA and HF.

## 5. Conclusions

In summary, our results support the idea of a causal relationship between UA and HF, but not of SA and HF. By TSMR, a significant positive causal relationship between UA and HF was demonstrated. Although the mechanism of association between UA and HF is not fully understood, the results of the current MR analysis suggest that UA may play a crucial role in the pathogenesis of HF. Therefore, we strongly recommend that patients with chronic liver disease developing angina, especially UA, should be treated aggressively for angina and monitored closely for their liver function. Once a significant or sustained deterioration in liver function is detected, prompt treatment should be instituted to minimize the risk of HF.

## Figures and Tables

**Table 1:** Characteristics of data used in the Mendelian randomization study.

Exposures /Outcome	GWAS ID	Ethnicity	Year	Observation Sample Size	Control Sample Size	Total Sample Size	Number of SNP
UA	ebi-a-GCST90018932	European	2021	9481	446,987	456,468	24,179,929
SA	ebi-a-GCST90018915	European	2021	17,894	325,132	343,026	19,057,124
HF	finn-b-K11_HEPFAIL	European	2021	464	213,592	214,056	16,380,437

**Table 2:** A total of 11 genome-wide significant SNPs were used as IVs to investigate the causal relationship between UA and HF.

No.	SNPs	CHR *	Position	EA *	OA *	Beta	SE *	F-Stat *	*p*-Value
1	rs10305838	4	148400256	C	T	0.130	0.016	68.246	1.28 × 10^−16^
2	rs11206803	1	56877509	T	C	0.070	0.013	31.092	2.61 × 10^−8^
3	rs11584234	1	115907009	G	T	−0.095	0.017	32.682	9.79 × 10^−9^
4	rs17637472	17	47461433	A	G	0.081	0.015	30.975	2.79 × 10^−8^
5	rs2240191	12	113335731	T	G	0.104	0.018	35.114	3.30 × 10^−9^
6	rs2288873	19	41852979	G	A	−0.070	0.013	30.641	3.35 × 10^−8^
7	rs28720373	4	148291242	T	C	−0.083	0.015	30.691	3.09 × 10^−8^
8	rs4409766	10	104616663	C	T	−0.108	0.017	39.261	3.30 × 10^−10^
9	rs7176070	15	79033489	C	T	−0.083	0.013	38.089	7.16 × 10^−10^
10	rs75346744	2	21520627	G	A	0.219	0.040	30.458	3.28 × 10^−8^
11	rs79780963	10	104952499	T	C	−0.105	0.018	33.898	5.53 × 10^−9^

* Abbreviations: CHR, chromosome; EA, effect allele; OA, other allele; SE, standard error; F-Stat, F-statistics.

**Table 3:** A total of 33 genome-wide significant SNPs were used as IVs to investigate the causal relationship between SA and HF.

No.	SNPs	CHR	Position	EA	OA	Beta	SE	F-Stat	*p*-Value
1	rs10305838	4	148400256	C	T	0.094	0.010	86.987	1.60 × 10^−20^
2	rs11072799	15	79027814	T	G	−0.075	0.009	70.001	7.15 × 10^−17^
3	rs11107908	12	95521242	T	C	−0.082	0.012	47.036	6.16 × 10^−12^
4	rs111876722	11	201922	C	T	0.089	0.015	34.350	4.13 × 10^−9^
5	rs11191593	10	104939215	C	T	−0.084	0.012	53.480	3.01 × 10^−13^
6	rs113429881	6	25064756	A	G	−0.121	0.021	31.917	1.69 × 10^−8^
7	rs114846969	19	11191197	A	G	−0.101	0.018	31.076	2.45 × 10^−8^
8	rs11600200	11	120334680	G	T	0.077	0.014	31.295	2.25 × 10^−8^
9	rs12928974	16	75304957	T	C	0.052	0.008	37.588	9.55 × 10^−10^
10	rs13031230	2	164912119	T	C	−0.060	0.009	42.751	7.45 × 10^−11^
11	rs17114036	1	56962821	G	A	−0.134	0.018	56.124	6.13 × 10^−14^
12	rs1893250	18	20002944	C	A	0.056	0.009	40.931	1.70 × 10^−10^
13	rs2166529	2	85742175	T	G	0.067	0.009	61.761	2.97 × 10^−15^
14	rs223288	6	57105602	C	T	−0.113	0.019	34.577	4.39 × 10^−9^
15	rs2306556	4	156638573	G	A	−0.064	0.010	39.863	2.48 × 10^−10^
16	rs2327429	6	134209837	C	T	−0.079	0.009	81.829	9.57 × 10^−20^
17	rs2820309	1	201834944	G	A	0.057	0.010	30.517	3.60 × 10^−8^
18	rs28451064	21	35593827	A	G	0.126	0.017	52.709	4.00 × 10^−13^
19	rs34767248	1	222793770	C	T	0.066	0.009	58.250	2.21 × 10^−14^
20	rs35058456	2	19943632	G	T	−0.056	0.009	41.432	1.45 × 10^−10^
21	rs3741380	11	65349063	A	G	0.048	0.008	32.926	8.11 × 10^−9^
22	rs4285828	10	44602514	C	T	−0.049	0.008	34.145	4.94 × 10^−9^
23	rs4778730	15	79253321	G	T	−0.066	0.011	33.721	6.17 × 10^−9^
24	rs4790881	17	2068932	A	C	0.051	0.009	33.983	6.17 × 10^−9^
25	rs62013193	15	79202362	T	C	0.065	0.011	32.884	8.68 × 10^−9^
26	rs6504606	17	47405349	G	A	0.056	0.010	31.882	1.53 × 10^−8^
27	rs6537862	1	115884184	A	G	−0.067	0.012	31.173	2.32 × 10^−8^
28	rs6694258	1	154428505	A	C	−0.047	0.008	31.387	2.29 × 10^−8^
29	rs7500448	16	83045790	G	A	−0.057	0.010	35.496	2.50 × 10^−9^
30	rs75041078	19	41877491	A	G	0.058	0.009	38.466	5.89 × 10^−10^
31	rs75346744	2	21520627	G	A	0.158	0.025	40.384	2.00 × 10^−10^
32	rs7681920	4	95937284	A	C	−0.057	0.009	41.368	1.34 × 10^−10^
33	rs79780963	10	104952499	T	C	−0.074	0.012	40.476	1.82 × 10^−10^

* Abbreviations: CHR, chromosome; EA, effect allele; OA, other allele; SE, standard error; F-Stat, F-statistics.

**Table 4:** Heterogeneity and pleiotropy analyses between UA, SA, and HF.

Exposures	Outcome	Cochrane’s Q				MR-PRESSO		Egger Intercept	
		IVW	*p*-Val *	MR Egger	*p*-Val	Outliers	*p*-Val	Intercept	*p*-Val
UA	HF	5.927	0.821	5.639	0.775	0	0.827	−6.41 × 10^−2^	0.604
SA	HF	25.862	0.770	25.719	0.735	0	0.722	−1.93 × 10^−2^	0.708

* Abbreviation: Val, value.

## Data Availability

The data that support the findings of this study are openly available in the OpenGWAS database (https://gwas.mrcieu.ac.uk/ (accessed on 19 October 2023)), reference number (ebi-a-GCST90018932; ebi-a-GCST90018915; finn-b-K11_HEPFAIL).

## References

[1] Manfredi R., Verdoia M., Compagnucci P., Barbarossa A., Stronati G., Casella M., Dello Russo A., Guerra F., Ciliberti G. (2022). Angina in 2022: Current Perspectives. J. Clin. Med..

[2] Schef K.W., Tornvall P., Alfredsson J., Hagström E., Ravn-Fischer A., Soderberg S., Yndigegn T., Jernberg T. (2023). Prevalence of angina pectoris and association with coronary atherosclerosis in a general population. Heart.

[3] Ahmed W., Muhammad T., Maurya C., Akhtar S.N. (2023). Prevalence and factors associated with undiagnosed and uncontrolled heart disease: A study based on self-reported chronic heart disease and symptom-based angina pectoris among middle-aged and older Indian adults. PLoS ONE.

[4] Heutinck J.M., de Koning I.A., Vromen T., Thijssen D.H.J., Kemps H.M.C. (2023). Exercise-based cardiac rehabilitation in stable angina pectoris: A narrative review on current evidence and underlying physiological mechanisms. Neth. Heart J..

[5] Byrne R.A., Rossello X., Coughlan J.J., Barbato E., Berry C., Chieffo A., Claeys M.J., Dan G.-A., Dweck M.R., Galbraith M. (2023). 2023 ESC Guidelines for the management of acute coronary syndromes: Developed by the task force on the management of acute coronary syndromes of the European Society of Cardiology (ESC). Eur. Heart J..

[6] Abbas A., Raza A., Ullah M., Hendi A.A., Akbar F., Khan S.U., Zaman U., Saeed S., Ur Rehman K., Sultan S. (2023). A Comprehensive Review: Epidemiological Strategies, Catheterization and Biomarkers used as a Bioweapon in Diagnosis and Management of Cardio Vascular Diseases. Curr. Probl. Cardiol..

[7] El Hadi H., Di Vincenzo A., Vettor R., Rossato M. (2020). Relationship between Heart Disease and Liver Disease: A Two-Way Street. Cells.

[8] Jain V., Ghosh R.K., Bandyopadhyay D., Kondapaneni M., Mondal S., Hajra A., Aronow W.S., Lavie C.J. (2021). Serum Bilirubin and Coronary Artery Disease: Intricate Relationship, Pathophysiology, and Recent Evidence. Curr. Probl. Cardiol..

[9] Zhang Y., Fang X.M. (2021). Hepatocardiac or Cardiohepatic Interaction: From Traditional Chinese Medicine to Western Medicine. Evid. Based Complement. Altern. Med..

[10] Lee W.M. (2022). Acute liver failure. Yamada’s Textbook of Gastroenterology.

[11] Khalil A.W., Iqbal Z., Adhikari A., Khan H., Nishan U., Iqbal A., Bangash J.A., Tarar O.M., Bilal A., Khan S.A. (2024). Spectroscopic characterization of eupalitin-3-O-β-D-galactopyranoside from Boerhavia procumbens: In vivo hepato-protective potential in rat model. Spectrochim. Acta Part A Mol. Biomol. Spectrosc..

[12] Rovegno M., Vera M., Ruiz A., Benítez C. (2019). Current concepts in acute liver failure. Ann. Hepatol..

[13] Br V.K., Sarin S.K. (2023). Acute-on-chronic liver failure: Terminology, mechanisms and management. Clin. Mol. Hepatol..

[14] Luo J., Li J., Li P., Liang X., Hassan H.M., Moreau R., Li J. (2023). Acute-on-chronic liver failure: Far to go—A review. Crit. Care.

[15] Weiler N., Schlotmann A., Schnitzbauer A.A., Zeuzem S., Welker M.W. (2020). The Epidemiology of Acute Liver Failure. Dtsch. Arztebl. Int..

[16] Cusack M.R., Marber M.S., Lambiase P.D., Bucknall C.A., Redwood S.R. (2002). Systemic inflammation in unstable angina is the result of myocardial necrosis. J. Am. Coll. Cardiol..

[17] Wang S., Zhu H., Pan L., Zhang M., Wan X., Xu H., Hua R., Zhu M., Gao P. (2023). Systemic inflammatory regulators and risk of acute-on-chronic liver failure: A bidirectional mendelian-randomization study. Front. Cell Dev. Biol..

[18] Laleman W., Claria J., Van der Merwe S., Moreau R., Trebicka J. (2018). Systemic Inflammation and Acute-on-Chronic Liver Failure: Too Much, Not Enough. Can. J. Gastroenterol. Hepatol..

[19] Fuhrmann V., Jäger B., Zubkova A., Drolz A. (2010). Hypoxic hepatitis—Epidemiology, pathophysiology and clinical management. Wien. Klin. Wochenschr..

[20] Waseem N., Chen P.H. (2016). Hypoxic Hepatitis: A Review and Clinical Update. J. Clin. Transl. Hepatol..

[21] Jonsdottir S., Arnardottir M.B., Andresson J.A., Bjornsson H.K., Lund S.H., Bjornsson E.S. (2022). Prevalence, clinical characteristics and outcomes of hypoxic hepatitis in critically ill patients. Scand. J. Gastroenterol..

[22] Sanderson E., Glymour M.M., Holmes M.V., Kang H., Morrison J., Munafò M.R., Palmer T., Schooling C.M., Wallace C., Zhao Q. (2022). Mendelian randomization. Nat. Rev. Methods Primers.

[23] Andreasen L. (2021). Mendelian randomization—A powerful tool to study the causal effects of atrial fibrillation on loss of brain volume. BMC Med..

[24] Birney E. (2022). Mendelian Randomization. Cold Spring Harb. Perspect. Med..

[25] Chen L., Yang H., Li H., He C., Yang L., Lv G. (2022). Insights into modifiable risk factors of cholelithiasis: A Mendelian randomization study. Hepatol..

[26] Lee Y.H. (2020). Overview of Mendelian Randomization Analysis. J. Rheum. Dis..

[27] Kain J., Owen K.A., Marion M.C., Langefeld C.D., Grammer A.C., Lipsky P.E. (2022). Mendelian randomization and pathway analysis demonstrate shared genetic associations between lupus and coronary artery disease. Cell Rep. Med..

[28] Zhu Q., Hua L., Chen L., Mu T., Dong D., Xu J., Shen C. (2023). Causal association between obstructive sleep apnea and gastroesophageal reflux disease: A bidirectional two-sample Mendelian randomization study. Front. Genet..

[29] Poelzl G., Ess M., Mussner-Seeber C., Pachinger O., Frick M., Ulmer H. (2012). Liver dysfunction in chronic heart failure: Prevalence, characteristics and prognostic significance. Eur. J. Clin. Investig..

[30] Stolz L., Kirchner M., Steffen J., Doldi P.M., Braun D., Weckbach L.T., Stocker T.J., Löw K., Fischer J., Haum M. (2023). Cardio-hepatic syndrome in patients undergoing transcatheter aortic valve replacement. Clin. Res. Cardiol. Off. J. Ger. Card. Soc..

[31] Kobalava Z.D., Villevalde S.V., Soloveva A.E. (2016). Cardio-hepatic Syndrome in Heart Failure: Prevalence, Pathogenesis and Prognostic Significance. Kardiologiia.

[32] Liang W., He X., Wu D., Xue R., Dong B., Owusu-Agyeman M., Zhao J., Cai L., You Z., Dong Y. (2021). Prognostic Implication of Liver Function Tests in Heart Failure With Preserved Ejection Fraction Without Chronic Hepatic Diseases: Insight From TOPCAT Trial. Front. Cardiovasc. Med..

[33] Møller S., Bernardi M. (2013). Interactions of the heart and the liver. Eur. Heart J..

[34] Wuthnow C., Bharwad A., Kamran S., Brake M., Rowe K. (2023). Ischemic Hepatitis and Acute Kidney Injury Following Cardioversion. Kans. J. Med..

[35] Van den broecke A., Van Coile L., Decruyenaere A., Colpaert K., Benoit D., Van Vlierberghe H., Decruyenaere J. (2018). Epidemiology, causes, evolution and outcome in a single-center cohort of 1116 critically ill patients with hypoxic hepatitis. Ann Intensive Care.

[36] Mukundan S.V., Marbach J.A. (2023). Live(r) and let die: Redefining hypoxic hepatitis in cardiogenic shock. Eur. Heart J. Acute Cardiovasc. Care.

[37] Henrion J. (2012). Hypoxic hepatitis. Liver Int..

[38] Omran S., Greiner A. (2023). Ischemic hepatitis due to an occlusion of visceral arteries: A case report. J. Surg. Case Rep..

[39] Metra M., Dinatolo E., Dasseni N. (2019). The New Heart Failure Association Definition of Advanced Heart Failure. Card. Fail. Rev..

[40] Murphy S.P., Ibrahim N.E., Januzzi J.L. (2020). Heart Failure With Reduced Ejection Fraction: A Review. JAMA.

[41] Hwang D., Park S.H., Koo B.K. (2023). Ischemia With Nonobstructive Coronary Artery Disease: Concept, Assessment, and Management. JACC Asia.

[42] Deng J., He J., Wang J., Cheng C.W., Jiao Y., Wang N., Li J., Wang P., Han F., Lyu A. (2023). Reporting quality of randomized controlled trials of angina pectoris with integrated traditional Chinese and western medicine interventions: A cross-sectional study. BMC Med. Res. Methodol..

[43] Larsson S.C., Butterworth A.S., Burgess S. (2023). Mendelian randomization for cardiovascular diseases: Principles and applications. Eur. Heart J..

[44] Dirchwolf M., Ruf A.E. (2015). Role of systemic inflammation in cirrhosis: From pathogenesis to prognosis. World J. Hepatol..

[45] Deng W., Farricielli L. (2013). Hypoxic hepatitis and acute liver failure in a patient with newly onset atrial fibrillation and diltiazem infusion. BMJ Case Rep..

[46] Theofilis P., Oikonomou E., Chasikidis C., Tsioufis K., Tousoulis D. (2023). Pathophysiology of Acute Coronary Syndromes—Diagnostic and Treatment Considerations. Life.

[47] Koehne de Gonzalez A.K., Lefkowitch J.H. (2017). Heart Disease and the Liver: Pathologic Evaluation. Gastroenterol. Clin. N. Am..

[48] Xanthopoulos A., Starling R.C., Kitai T., Triposkiadis F. (2019). Heart Failure and Liver Disease: Cardiohepatic Interactions. JACC Heart Fail..

